# Carvacrol Ameliorates Pathological Cardiac Hypertrophy in Both *In-vivo* and *In-vitro* Models

**DOI:** 10.22037/ijpr.2019.1100766

**Published:** 2019

**Authors:** Mohabbat Jamhiri, Farzan Safi Dahaj, Akram Astani, Seyed Hasan Hejazian, Zeynab Hafizibarjin, Mojtaba Ghobadi, Ali Moradi, Arezu Khoradmehr, Fatemeh Safari

**Affiliations:** a *Department of Physiology, Faculty of Medicine, Shahid Sadoughi University of Medical Sciences, Yazd, Iran.*; b *Department of Microbiology, Faculty of Medicine, Shahid Sadoughi University of Medical Sciences, Yazd, Iran.*; c *Department of Biochemistry, Faculty of Medicine, Shahid Sadoughi University of Medical Sciences, Yazd, Iran.*; d *Yazd Reproductive Sciences Institute, Shahid Sadoughi University of Medical Sciences, Yazd, Iran.*; e *Biotechnology Research Center, International Campus, Shahid Sadoughi University of Medical Sciences, Yazd, Iran.*

**Keywords:** Cardiac hypertrophy, Carvacrol, Rat, H9c2 cells, Blood pressure, Antioxidant, Apoptosis

## Abstract

Hypertension-induced left ventricular hypertrophy is the most important risk factor for heart failure. This study aimed at investigating the effects of monoterpenoid phenol, carvacrol, on myocardial hypertrophy using both *in-vivo* and *in-vitro* models. Male Wistar rats were divided into the control (Ctl), un-treated hypertrophy (H), and carvacrol-treated hypertrophy groups (25, 50 and 75 mg/kg/day, Car+H). In the hypertrophy groups animals underwent abdominal aorta banding. Blood pressure (BP) was recorded via carotid artery cannulation. TUNEL assay and Masson’s trichrome staining were used to assess apoptosis and fibrosis, respectively. The 2-2-diphenyl 1-picril-hydrasil )DPPH( radical scavenging activity and malondialdehyde (MDA) level were estimated by biochemical tests. In *in-vitro *study H9c2 cardiomyoblasts were treated with angiotensin II (Ang II) to promote hypertrophy. Cell size was measured using crystal violet staining. Gene expression was evaluated by real-time RT*-*PCR technique. In the carvacrol-treated rats BP, heart rate, and heart weight to the body weight ratio were significantly decreased. *In-vitro *study showed that H9c2 cell size was significantly reduced compared to Ang II-treated cells. Both *in-vivo* and *in-vitro *studies demonstrated that carvacrol decreased atrial natriuretic peptide )ANP( mRNA level significantly (*vs.* H groups). The number of apoptotic cells increased in H group, while it was decreased in the Car50+H and Car75+H. In Car+H groups, in comparison with H group, the serum concentration of MDA was decreased and DPPH was increased significantly. Our findings demonstrated that carvacrol decreases hypertrophy markers in *in-vivo *and *in-vitro *models of hypertrophy.

## Introduction

Left ventricle hypertrophy (LVH) is considered as an adaptive response to pathological volume or pressure stresses in order to adjust pump function to the circulatory demands. LVH is identified by the increase of cardiomyocyte′s size that is characterized by re-expression of fetal genes, enhanced protein synthesis, impaired myocardial vascularization, and extra cellular matrix alteration (1). 

Among the factors leading to LVH, hypertension is considered as the main biomechanical stress. Even though pressure overload-induced LVH is a compensatory response to improve the heart function and to normalize cardiac output, sustained LVH represents the main predictor of adverse cardiovascular prognosis in hypertensive patients. Indeed, LVH is the main risk factor for heart failure which is a leading cause of mortality worldwide (2, 3). 

At the cellular and molecular levels, the severity of LVH-induced cardiac dysfunction correlates strongly with overproduction of reactive oxygen species (ROS) defined as oxidative stress (4, 5). 

The main sources of ROS in cardiomyocytes are mitochondria, xanthine oxidase and NADPH oxidases (6-8). It has been reported that the increased generation of ROS is the key contributor in hypertrophic effect mediated by Angiotensin II, endothelin-1, TNF-α and alpha-adrenergic agonists (9). Oxidative stress can activate apoptotic factors such as proapoptotic members of BCL-2 family and caspases. Experimental studies revealed that transition from adaptive to maladaptive hypertrophy is accompanied by cardiomyocytes loss due to apoptosis. 

The complex molecular signaling regulating cardiomyocytes′ growth also enhances myocytes′ survival; however, as soon as the LVH proceeds, the increased production of ROS and some of the mediators such as Ang II, inflammatory interleukins, and catecholamines promote over production/activation of pro-apoptotic factors, which leads to apoptosis (10, 11). 

Despite valuable therapeutic advances, the morbidity and mortality of heart failure continue to remain unacceptably high. Therefore, finding the new therapeutic agent remains an unmet need. Clinical end experimental studies have demonstrated that active herbal extracts and components like monoterpenes could have a great role in cardiovascular resistance against diseases (12). Carvacrol (C₆H₃CH₃) is a monoterpenoid phenol isomer present in essential oil of *genera Origanum*, *Thymus*, *Coridothymus*, *Thymbra*, *Satureja* and *Lippia* (13). Results of previous research have shown diverse pharmacological properties of carvacrol such as antioxidant, antimicrobial, antiproliferative, antispasmodic, and anti-inflammatory effects (14, 15). Although these protective effects have been identified, little is known about the possible effects of this phenol in heart and vessels. Carvacrol protects the heart against myocardial ischemia-reperfusion injury and acute myocardial infarction in animal models (16, 17). Carvacrol reduces blood pressure and heart rate in normotensive rats and prevents L-NAME induced hypertension (18). This monoterpen causes an endothelium-independent aortic relaxation through calcium influx inhibition (19). In addition, carvacrol inhibits L-type Ca2^+^ current in canine and human ventricles cardiomyocytes (20). Endothelium-dependent vasorelaxation effect of carvacrol in cerebral artery of rats has been also shown (21). However, there is no report on the possible protective effects of carvacrol against cardiac hypertrophy. Therefore, the current study aims to investigate the effects of carvacrol on LVH markers including blood pressure, heart weight to body weight ratio, fibrosis and natriuretic peptide mRNA levels in animal model of pressure-overload induced hypertrophy. Furthermore, this study aims at evaluating the apoptosis and oxidative stress-related parameters and investigating the direct effect of carvacrol on Ang II–induced hypertrophy in H9c2 cardiomyoblasts.

## Experimental

*Experimental Design*

This study was conducted on male Wistar rats (170-210 g). The animals were housed at the animal house under diurnal cycle of 12 h light/12 h darkness at an approximate temperature of 22-25 °C. Rats received standard chow and water ad libitum. The experimental protocols were approved by the Animal Ethics Care and Use Committee of Shahid Sadoughi University of Medical Sciences. The animals were divided into the seven experimental groups (n = 10).

1. Ctl group: Intact animals served as control.

2. H group: To induce hypertrophy model, animals underwent abdominal aorta banding.

3. Car25+H group

4. Car50+H group

5. Car75+H group

The treated groups (3, 4, and 5) received carvacrol (Car, Purity ≥98%- Sigma- Aldrich, USA), at doses of 25, 50 and 75 mg/kg/day (ip). Treatment had been started seven days before induction of hypertrophy and continued for three weeks. 

6. DMSO+H group: Since carvacrol was dissolved in 4% dimethyl sulfoxide [DMSO], this group of animals, as the sham, received DMSO.

Car50 group: the rats were given carvacrol (50 mg/kg/day), without hypertrophy induction. 

7. In all experimental groups blood pressure was recorded directly via carotid artery cannulation.

*Rat model of pressure overload-induced hypertrophy*

The rats were anesthetized by the intraperitoneal injection of ketamine (70-90 mg/kg) and xylazine (10 mg/kg). An incision was made in the left flank. After exposing the abdominal aorta a 21-guage needle was placed beside the artery and the suture was tied around it, then the needle was removed and abdominal wall muscles and skin were sutured. Three weeks after abdominal aorta stenosis, the myocardial hypertrophy model was induced due to chronic hypertension. At the end of the third week, the left carotid artery was cannulated to record the blood pressure directly. Then, the hearts were weighed to estimate the ratio of the heart weight (HW) to the body weight (BW). To separate the serum, the blood samples were drawn from atrium and centrifuged at 3000 rpm for 20 min at room temperature. The animal serums were kept at -80 ºC for biochemical studies. Finally, the heart was excised quickly through thoracotomy, the left ventricle tissue was separated and placed in liquid nitrogen for molecular studies. 

Abdominal aorta banding in rats, is a reproducible model of cardiac hypertrophy which has been commonly used for studying pressure overload-induced LVH. Our previous study along with other studies has shown that blood pressure increases significantly within 3-4 days after aortic banding, and over time, this chronic hemodynamic overload leads the hypertrophy response in the left ventricle (22, 23).

*Histological study*

To assess the cardiac fibrosis in experimental groups, three samples of each group were fixed in 10% formaldehyde. After tissue processing and paraffin embedding, the cross-sectional slices were stained with Masson trichrome for collagen deposition assessment. In this staining technique, the nucleus appears in dark blue, while the cytoplasm is light scarlet, the muscle is dark scarlet, and also, the collagen is green or blue.

*Cell culture and Angiotensin II-induced hypertrophy model*

Rat myocyte H9c2 cell series were obtained from the Pasteur Institute-Iran, and were serially passaged in DMEM (Dulbecco′s Modified Eagle′s Medium, Gibco®) made up of 10% fetal bovine serum (FBS) as a supplement, 100 U/mL penicillin and 100 μg/mL streptomycin. The cells were seeded onto 6-well plates at a density of 1 × 10^5^ cells/well and incubated in 37 °C with humidified atmosphere of 5% CO_2_. The cell cultures between passages 5 to 7 were used for the experiments. The cells treated with 0.01 to 100 μmol of carvacrol for 24 h. The cell viability was evaluated using MTT test. Based on the results of MTT, the cells pretreated with 0.01, 0.1, and 1 μmol of carvacrol to evaluate its preventive effect on cardiac hypertrophy. Twenty-four hours after carvacrol pretreatment, angiotensin II (Sigma®, A9525, 1 μmol/L) was added to induce hypertrophy model.

*Cell size measurement*

In order to measure H9c2 cells area, the cells were fixed with 10% formaldehyde for 1-2 min, and stained with crystal violet solution. After irrigating the plate with water, the cells were observed using a microscope (Zeiss) and the images were captured at 10X magnification (Sony, Syber-shot, DSCWX200 camera). The cell area was measured applying ImageJ software 1.49v. (Parlee *et al.* 2014). In each group, at least 150 cells were measured.

**Table 1:** Primers sequences used in this study

**Gene**	**Forward primer (5'–3')**	**Reverse primer (5'–3')**
ANP	GAGGAGAAGATGCCGGTAG	CTAGAGAGGGAGCTAAGTG
BNP	TGATTCTGCTCCTGCTTTTC	GTGGATTGTTCTGGAGACTG
B-actin	GAACCCTAAGGCCAACCGTGAA	ATAGCAGCCACAAAAAGGGAAA

**Table 2:** Hemodynamic parameters among experimental groups

**Groups**	**SP (mmHg)**	**DP (mmHg)**	**HR (beats/min)**
Ctl	102.8 ± 6	81.75 ± 6	292 ± 14
H	153 ± 5.7***	128.8 ± 2***	271 ± 11
DMSO+H	140 ± 7*	116 ± 5.6***	259 ± 8
Car25+H	106.7 ± 5###	61.2 ± 6###	236 ± 16**
Car50+H	116.3 ± 6.5#	92.5 ± 5##	229 ± 17*
Car75+H	100.8 ± 6###	65.75 ± 4###	239 ± 18*
Car50	98.2 ± 5	72.2 ± 5.4	243 ± 15*

Systolic blood pressure (SP), diastolic blood pressure (DP) and heart rate (HR) changes in the control (Ctl), hypertrophy (H), and carvacrol (Car+H) treated groups. Animals were treated with 25, 50 and 75 mg/kg/day of carvacrol. The data are displayed as mean ± SEM. *P < 0.05, **P < 0.01 and ***P < 0.001 *vs. *Ctl. #*P < *0.05, ##*P < *0.01 and ###*P < *0.001 *vs. *H group (n = 10).

**Figure 1:**
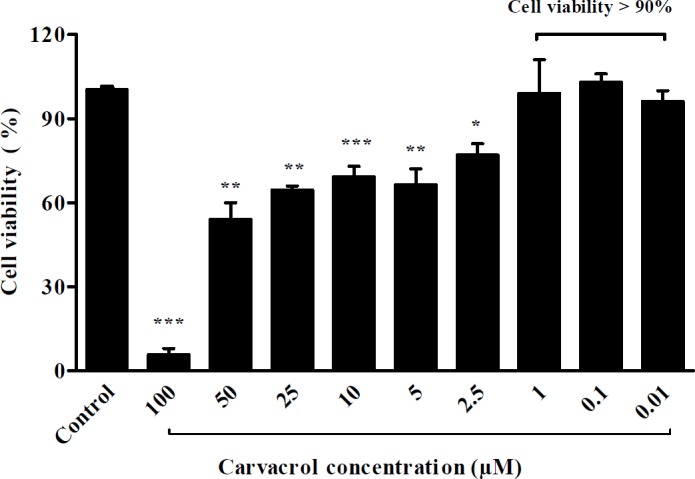
Assessment of H9c2 cells viability after treatment with different concentrations of carvacrol. Untreated cells served as control. Cell viability was evaluated by MTT test. Data are expressed as mean ± SEM.^ *^P < 0.05, ^**^P < 0.01 and ^***^P < 0.001 *vs.* control

**Figure 2:**
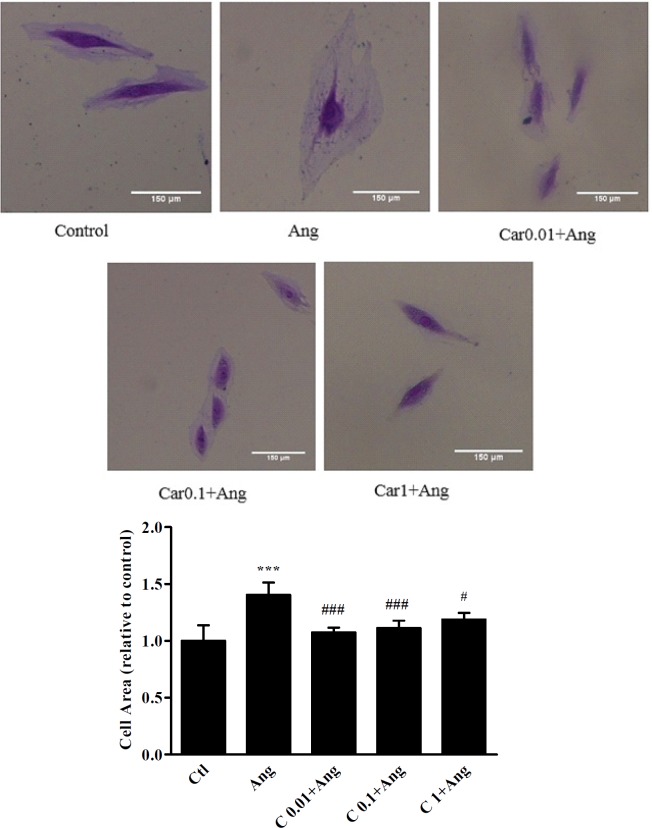
H9c2 cells size after treatment with different concentrations of carvacrol. The images show the cell size in un-treated (Ctl) and angiotensin II treated H9c2 cells (Ang, 1 μM) in the presence or absence of carvacrol. A significant decrease in cell size was observed when hypertrophied cells were pretreated with 0.01, 0.1 and 1 μM of carvacrol. Data are expressed as mean ± SEM.^ ***^P < 0.001 *vs.* Ctl,^ #^*P < *0.05 and ^###^*P < *0.001 *vs.* Ang

**Figure 3:**
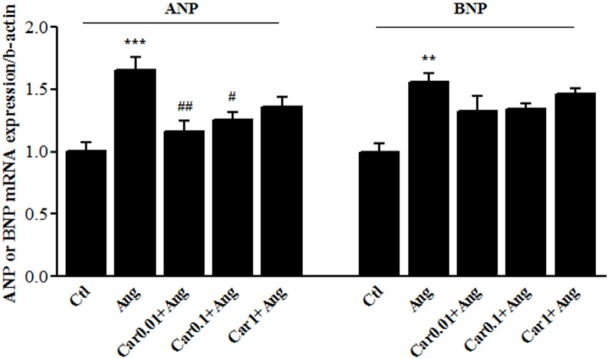
Transcription level of natriuretic peptides in H9c2 cells. Atrial and brain Natriuretic peptides (ANP and BNP, respectively) mRNA levels were assessed in the Angiotensin II (Ang)-induced hypertrophied H9c2 cells in the presence or absence of carvacrol (Car) at different concentrations. Un-treated cells served as control (Ctl). The data are displayed as mean ± SEM. ^**^P < 0.01 and ^***^P < 0.001 *vs.* Ctl. ^#^*P* < 0.05 and ^##^*P* < 0.01 *vs.* Ang

**Figure 4:**
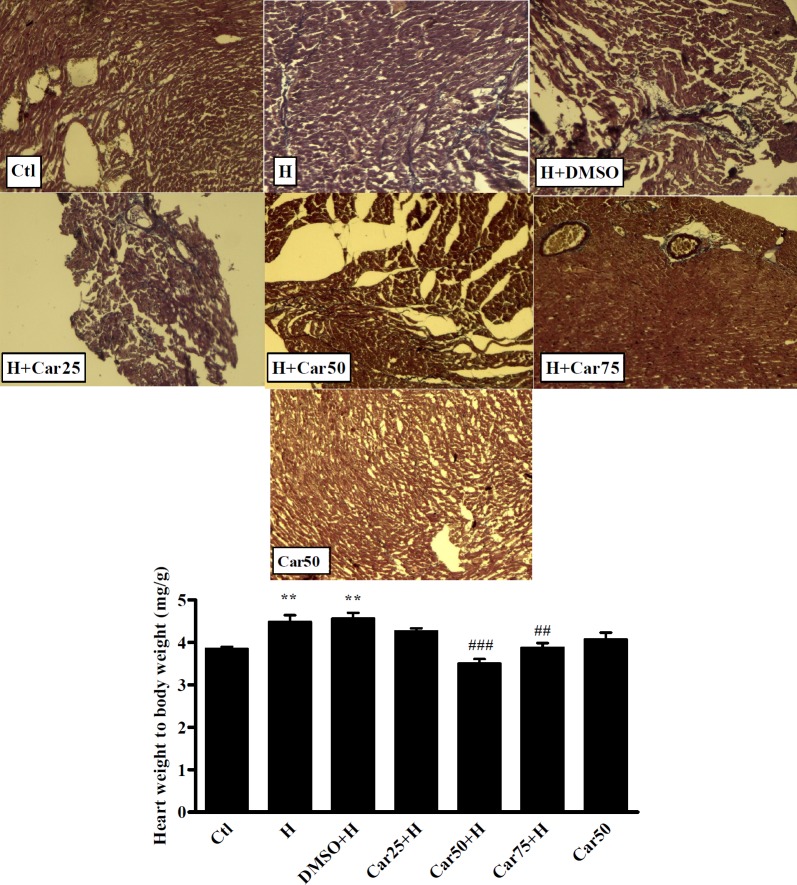
The heart weight to body weight ratio (HW/BW) in experimental groups. As an important marker of cardiac hypertrophy, HW/BW ratio was measured in the control (Ctl), hypertrophy (H), and carvacrol (Car+H) treated groups. DMSO used as the solvent. Animals were treated with 25, 50 and 75 mg/kg/day of carvacrol for three weeks. The data are displayed as mean ± SEM. ^**^P < 0.01 *vs.* Ctl. ^##^*P <* 0.01 and ^###^*P <* 0.001 *vs.* H group (n = 10). The top panel shows representative photomicrographs of Masson's Trichrome stained heart sections confirms fibrosis in un-treated hypertrophied hearts (20x magnification)

**Figure 5.**
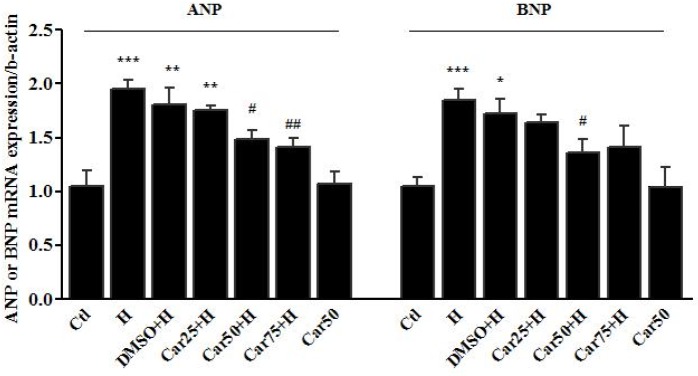
Cardiac transcription of natriuretic peptides. Atrial and brain Natriuretic peptides (ANP and BNP, respectively) mRNA levels were assessed in the left ventricular tissue of rats subjected to pressure overload-induced hypertrophy (H) in the presence or absence of carvacrol (Car) at different concentrations. Intact animals served as control (Ctl). DMSO used as the solvent of carvacrol. The data are displayed as mean ± SEM. ^*^P < 0.05, ^**^P < 0.01 and ^***^P < 0.001 *vs.* Ctl group. ^#^*P <* 0.05 and ^##^*P <* 0.01 *vs.* Ang or H group

**Figure 6:**
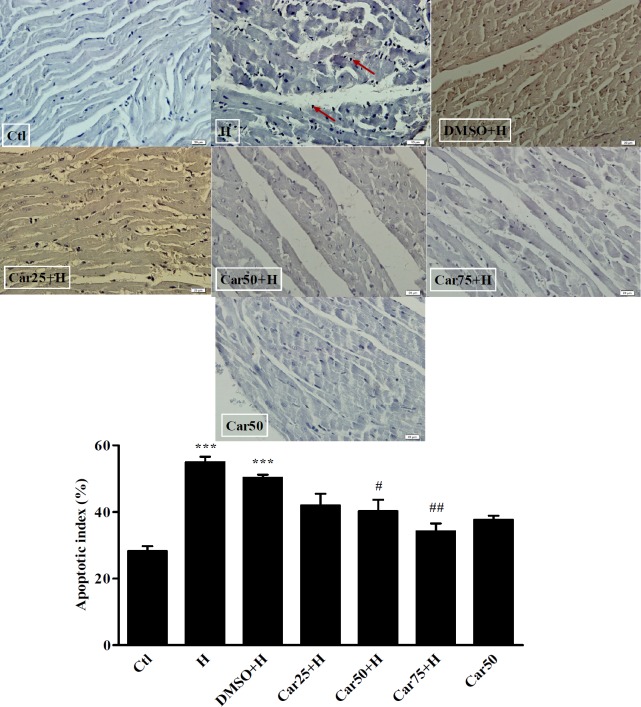
TUNEL staining and the apoptotic rate of cardiomyocytes. Cardiomyocytes apoptosis (arrows) was detected in untreated (H) and carvacrol (Car+H) treated rats subjected to aortic banding-induced hypertrophy. Intact animals served as control (Ctl). DMSO used as the solvent of carvacrol. Animals were treated with 25, 50 and 75 mg/kg/day of carvacrol for three weeks. In H group the apoptotic index was increased significantly. In Car50+H and Car75+H groups it was far less than that in the H group. Data are expressed as mean ± SEM.^ ***^P < 0.001 *vs.* Ctl, ^#^*P <* 0.05 and ^##^*P <* 0.01, *vs.* H group

**Figure 7:**
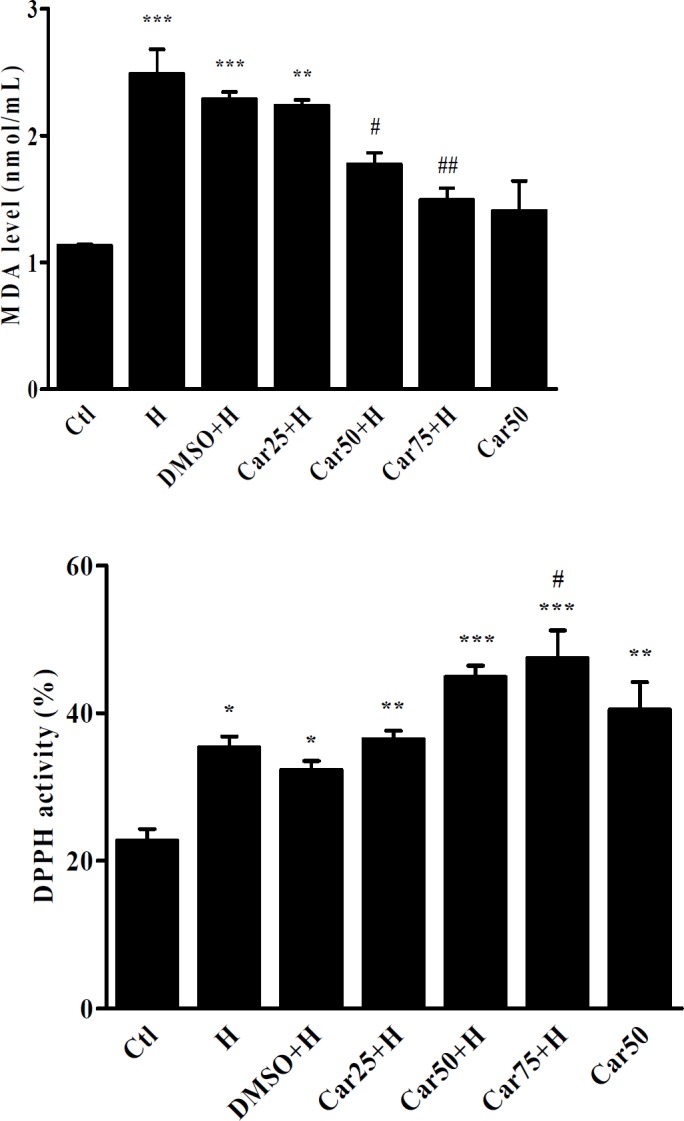
Serum concentration of Malondealdehyde (MDA) and 2-2-diphenyl 1-picril-hydrasil (DPPH) radical scavenging activity. MDA and DPPH activity were measured in the serum of untreated (H) carvacrol (Car+H) treated rats subjected to aortic banding-induced hypertrophy. Intact animals served as control (Ctl). DMSO used as the solvent of carvacrol. Animals were treated with 25, 50 and 75 mg/kg/day of carvacrol for three weeks. Data are expressed as mean ± SEM.^ *^P < 0.05,^ **^P < 0.01,^ ***^P < 0.001 *vs.* Ctl, ^#^*P <* 0.05 and ^##^*P <* 0.01, *vs.* H group

*MTT (3-[4, 5-dimethylthiazol-2-yl]-2, 5-diphenyl-tetrazolium bromide) assay*

To assess the effect of carvacrol on H9c2 cells viability, the sub confluent cells were treated with different concentrations of carvacrol (100, 50, 25, 10, 5, 2.5, 1, 0.1, and 0.01 μmol/L) diluted from carvacrol stock (66000 μmol/L dissolved in DMSO) for twenty-four hours. After treatment with drug, the medium was replaced by 20 μL of MTT (5 mg/mL dissolved in PBS) per well incubated in 37 °C for three hours. After removing MTT from wells, 150 μL of DMSO (Cinagen®, Iran) was added to each well. Optical density was read at 570 and 630 nm on a multi well ELISA reader (Biotech Instrument Model- Box998). The percentage of cell viability was measured in comparison with the control group.

*RNA Isolation and real time RT-PCR assay*

The tissue of left ventricle and the cellular samples were lysed by RNX-plus solution (Sinagen, Iran) and homogenized (T10Bhomogenizer- Germany). 

The extraction processes were followed according to the manufacturer′s instructions. To estimate the RNA concentration with spectrophotometry using the nanodrop set (Biotech Instrument Model- Box998), a concentration of the sample was read at 260 nanometer wavelength. The extracted RNA was immediately used in cDNA synthesis or preserved in -80 ºC freezer. To prepare cDNA, the reverse transcription reaction was performed using RevertAid™M-MuLV Reverse transcriptase (Fermentas, USA). After optimization of the reaction, the cDNAs pertaining to the experimental groups were obtained by MasterMix containing cyber green (Takara) and specific primers under RT-PCR reaction. The sequence of the primers was used is summarized in Table 1. 

The Beta-actin gene was considered as the reference gene. The ratio of expression of the target gene to the reference gene was estimated according to 2^-ΔΔct^ method.

*Antioxidant capacity of carvacrol by the use of DPPH*

The antioxidant effect of carvacrol was assessed by 2-2-diphenyl 1-picril-hydrasil (DPPH Merck, Germany). DPPH solution (400 µL), phosphate buffer (380 µL, pH 7.4) and serum (20 µL) were mixed and placed at ambient temperature for 30 min. The samples absorption was read by nanodrop set (Biotech Instrument Model- Box998) at 517 nanometer wavelength (24) .DPPH along with methanol and phosphate buffer were used as control. The antioxidant capacity of carvacrol was calculated with DPPH using the following formula:

DPPH radical scavenging activity% = [(OD_control_ – OD_sample_) ÷ OD_control_] × 100

*Malondialdehyde Measurement*

To measure the serum MDA level, 100 µL of serum, 5 µL of butilated toluene hydroxide, and 400 µL of 5% triclorostic acid were mixed and the prepared solution was centrifuged for 10 min at 300 rpm. After removing 200 µL of the obtained solution, 150 µL of thiobarbitoric was added to it and the mixture was placed in 95 ºC for 1 h. Then, it was preserved at -4 ºC and the absorption rate was measured at 532 and 572 nanometer wavelengths (25). To determine the concentration of MDA, tetroxypropan was used as control to plot the standard curve. 

*TUNEL assays*

Three slides from each heart were evaluated for cardiomyocytes apoptosis detection using a TUNEL assay kit (TUNEL Assay, Roche Applied Science) according to the manufacturer′s instructions. Briefly, the cardiac tissue slices deparaffinized in xylenes and rehydrated at gradient concentration ethanol. Proteinase k was added to increase membrane permeability. 

TUNEL mix and PI (Propidium iodide) were used for labeling and tracing. After washing, the slices were examined with the light microscope at 400x magnification. The numbers of total and TUNEL-positive cells were counted in each field. The results were expressed as apoptotic index (number of TUNEL-positive myocytes/total myocytes) × 100%. 

*Statistical analysis*

Data are presented as mean ± standard error of mean (SEM). Hemodynamic values were analyzed by the Kruskal–Wallis test with Dunn’s post-test for multi­ple comparison. Other statistical significances were evaluated using One-way analysis of variance (ANOVA), followed by Tukey′s multiple comparison post-hoc test. Statistical significance was designated at *P <* 0.05. Statistical analysis was performed using Prism-5 software. 

## Results

*In-vitro study*

*Effect of carvacrol pretreatment on H9c2 cell viability*

Before investigation of the preventive effect of carvacrol against hypertrophy, the cytotoxicity of various concentrations of carvacrol was tested by the MTT assay. In H9c2 cells, which had been treated with 0.01, 0.1 and 1 μM of carvacrol, the cell viability was more than 90% (Figure 1).

*Effect of carvacrol pretreatment on H9c2 cell size*

As shown in Figure 2, cell size analysis of H9c2 cells revealed that in Ang group cell size increased by 38 ± 10.5% in comparison with the control cells (*P* < 0.001). A significant decrease in cell size was observed when hypertrophic cells were pretreated with 0.01, 0.1, and 1 μM of carvacrol in comparison with Ang treated cells (*P* < 0.001, *P* < 0.001, and *P* < 0.05, respectively).

*Effects of carvacrol on the ANP and BNP mRNA levels in angiotensin-treated H9c2 cell*

Enhanced transcription level of ANP and BNP is a main marker of cardiac hypertrophy. The results of the *in-vitro *study showed that Ang II emphatically precipitated ANP mRNA level up to 65.5 ± 8.5% (*P* < 0.001 *vs.* Ctl). ANP mRNA level was increased in Car-0.01+Ang group by 16 ± 5.5% and in Car-0.1+Ang group by 25 ± 4% which shows a significant difference in comparison with Ang group (*P* < 0.01 and *P* < 0.05 respectively).

BNP gene expression was significantly increased after treatment of H9c2 cells with Ang II (P < 0.01, *vs.* Ctl), whereas it did not significantly change among carvacrol-treated groups (Figure 3).

*In-vivo study*

*Effect of carvacrol on blood pressure and HW/BW following aortic banding-induced myocardial hypertrophy*

As shown in Table 2, in the H group, systolic and diastolic blood pressure were increased significantly in comparison with Ctl group (*P <* 0.001). In all carvacrol treated groups, the systolic and diastolic blood pressure was significantly decreased compared to the H group. 

It was interesting that in H group, heart rate did not change significantly, while in Car25+H, Car50+H, and Car75+H groups the heart rate decreased significantly compared to the Ctl group (*P <* 0.01, *P <* 0.05 and *P <* 0.5 respectively). Also, in Car50 group in which animals received carvacrol without induction of hypertrophy, the heart rate decreased significantly (*P <* 0.01 *vs*. Ctl). 

The mean ratio of HW/BW increased in the H group to 4.8 ± 0.4 which shows a significant different with Ctl group (Ctl 3.59 ± 0.35, *P <* 0.001). 

Regarding treated groups, in the Car50+H and Car75+H groups the HW/BW ratio decreased to3.50 ± 0.3 and 3.88 ± 0.32 which was significant in comparison with H group (*P <* 0.001 and *P <* 0.01, respectively). Induction of hypertrophy was associated with fibrosis which was confirmed using Masson’s Trichrome staining (Figure 4).

*Effects of carvacrol on the ANP and BNP mRNA level in left ventricular tissue of hypertrophied hearts*

*In-vivo *study demonstrated that following pressure overload-induced hypertrophy, the cardiac level of ANP mRNA level was increased by 95.4 ± 8% in the H group (*P <* 0.001 *vs.* Ctl). ANP mRNA level was decreased in Car50+H group by 48.6 ± 9% and in Car75+H group by 41.2 ± 8.6%, indicating a significant change compared to the H group (*P <* 0.05 and *P <* 0.01, respectively). 

The transcription level of BNP increased by 84.5 ± 10.4% in the H group compared to the Ctl group, indicating a significant change (*P <* 0.001). Among the treatment groups, the level of BNP mRNA reached 35.9 ± 5% in the Car50+H group showing a significant decrease compared to the H group (*P <* 0.05) (Figure 5).

*Effect of carvacrol on the apoptotic index in the in left ventricular tissue of hypertrophied hearts*

Due to the importance of apoptosis in the pathogenesis of LVH, the rate of apoptosis was evaluated in experimental groups using TUNEL assay. As shown in Figure 6, the percentage of apoptotic cells has increased significantly in the H group (36.5 ± 3.9%) compared to the Ctl group (*P <* 0.001). Apoptotic index was reached to 26 ± 3.1% in the Car50+H group and to 16.25 ± 4% in Car75+H group. The result was significant compared to the H group (*P <* 0.05 and *P <* 0.01, respectively).

*Effect of carvacrol on DPPH radical scavenging activity in rats subjected to aortic-banding*

The DPPH radical scavenging activity of carvacrol was shown in Figure 7. In H and carvacrol-treated groups, DPPH radical scavenging activity was significantly increased in comparison with control group. It was interesting that in Car50 group in which the rats received carvacrol, without induction of hypertrophy. Increase of serum DPPH radical scavenging activity in Car75+H group was also significant compared with the H group (*P <* 0.05).

*Effect of carvacrol on malondealdehyde level in rats subjected to aortic-banding*

As shown in Figure 7 the serum concentration of MDA has significantly increased in the H group compared to the Ctl group (*P <* 0.001). In the Car50+H and Car75+H groups, MDA levels was significantly decreased in comparison with the H group (*P <* 0.05 and *P <* 0.01, respectively).

## Discussion

The results of the first part of the study indicated that following abdominal aortic banding, the cardiac hypertrophy markers such as blood pressure, HW/BW ratio, collagen deposition, ANP and BNP mRNA levels, and apoptotic rate were increased. These findings are consistent with the results shown in previous studies (26, 27). The results of the second part of the study revealed that the treatment of rats with carvacrol at doses of 50 and 75 mg/kg/day prevented the abdominal aortic banding-induced hypertension leading to a decrease in HW/BW ratio. This effect was associated with decreased level of ANP mRNA. 

Our results have shown that carvacrol at 25 mg/kg/day for 21 days could not exhibit anti-hypertrophic effects. Our data is in agreement with a study by Chen *et al.* demonstrating that carvacrol at 25 mg/kg/day could not protect the heart against ischemia reperfusion injury. But, increase the dose to 50 and 100 mg/kg ameliorated ischemia-induced heart injury by increase of superoxide dismutase and catalase activity (28). However, El-Sayed *et al. *in a recent study have demonstrated that carvacrol at 25 mg/kg/day for 16 days protected the heart against doxirubicine-induced cardiac toxicity (29). Different results may be attributed to several factors such as using the different models of cardiovascular diseases. 

In a pilot study we have shown that the dose of 100 mg/kg of carvacrol decrease baseline blood pressure significantly, therefore this dose was omitted from the study. It should be pointed that in our study, carvacrol decreased heart rate even in normal animals without induction of hypertrophy. The hypotensive and bradycardia effect of different doses of carvacrol (1, 5, 10 and 20 mg/kg i.v.) in non-anesthetized normotensive rats was reported recently (30). Therefore, a more detailed study is required for in-depth understanding the mechanisms and outcomes of the negative chronotropic effect of carvacrol. 

The fundamental question that needs to be considered is the possible mechanisms of preventive effect of carvacrol on cardiac hypertrophy, which is reported in the present study. In the *in-vivo *model, carvacrol dramatically prevented aortic banding-induced hypertension, so it could be concluded that preventive effect of carvacrol is directly linked to its hypotensive effect. Previous studies suggested that one of the main mechanisms involved in the hypotensive effect of carvacrol is the inhibition of different transient receptor potential (TRP) channels and voltage-dependent calcium channels. Ca^2+^ channels have been implicated in the signaling pathway that leads to cardiac hypertrophy. 

The TRP channels are a group of ionic channels found mostly in plasma membranes of various mammal cells and are responsible for the flux of cations. Several reports have demonstrated that hypertrophic agonists connected to G-protein coupled receptors activate Ca^2+^ entry through transient receptor potential canonical (TRPC) channels. The studies conducted on these channels indicated that TRPM4, TRPC1, TRPC3, and TRPC6 channels play an important role in the creation and progression of hypertrophy and hypertension (28, 29). 

Increased expression of TRPC3 and TRPC5 channels in the hypertrophied and failed hearts indicates that the TRPC channels may contribute to myocardial hypertrophy pathogenesis through calcium signaling and calcineurin/NFAT path activation (30). 

The study by Peixoto-Neves *et al.* conducted on rat aorta showed that carvacrol causes the endothelium-independent relaxation of aorta. Their study suggested that the inhibition of calcium current may be the mechanism behind the pharmacological effects of carvacrol (19). 

Additionally, Dantas *et al.* revealed that carvacrol induces endothelium-independent relaxation in loops of upper mesenteric vessels by inhibiting Ca^+2^ influx through receptor operated channels (ROC), store operated channels (SOC), and TRPC channels (31). Due to the inhibiting effect of carvacrol on the TRPC channels, it is possible that some part of the cardioprotective effects of carvacrol is because of peripheral vasodilation exerted through these channels. This hypothesis can be addressed in the future works. This study also demonstrated that carvacrol reduces heart rate even in normotensive rats. Therefore, it could be concluded that some parts of the hypotensive effect of carvacrol is probably due to bradycardia.

The current study revealed that carvacrol prevents cardiac hypertrophy; so, it may be hypothesized that this effect of carvacrol can be linked to its hemodynamic effects. To investigate this hypothesis, this study examined the direct effect of carvacrol on cellular model of hypertrophy. 

The results revealed that carvacrol prevents the angiotensin-induced hypertrophy of H9c2 cells. In other words, carvacrol exerts anti hypertrophy effects on cardiomyocytes independent of hemodynamic effects. Identifying the cellular and molecular mechanisms, which results in the effect of carvacrol on cardiomyocytes growth, can provide new insights into the basis of cardioprotective effects of this phenol. 

Another finding of the current study was that the apoptotic index increases during myocardial hypertrophy in the left ventricular tissue and the rate of apoptosis in hypertrophied hearts is decreased by administration of carvacrol. Apoptosis is a principal cause of cardiomyocytes loss in ischemia/reperfusion injury, hypertrophy and cardiac failure. Cardiac hypertrophy can be adjusted in the initial stages, while during progression it will be accompanied by an increased myocyte apoptotic index that leads to reduced cardiac performance gradually.

Increasing evidence suggests that hypertrophic signaling pathways cannot control the balance between myocyte death and life because of the decrease of survival and anti-apoptotic factors (such as Bcl-2, Bcl-xL, MnSOD and phosphoinositide 3-kinase (PI3K)–Akt signaling) and the increase of pro-apoptotic molecules (including Bax, Bad, cytochrome c, procaspases and active caspases). The oxidative stress is the main factor that triggers apoptosis in cardiomyocytes (11, 32). 

There is little report on the anti-apoptotic effects of carvacrol. Yu *et al.* demonstrated that carvacrol at doses of 25, 50 and 100 mg/kg/day exhibited anti-oxidative and anti-apoptotic effects in infracted myocardium through suppression of caspase-3 and pro-apoptotic factor BAX and upregulation of anti-apoptotic protein Bcl-2 (17). 

In studying the protective effects of carvacrol on cerebral focal ischemia it was indicated that 50 mg/kg of carvacrol decreased the infarct size and improved neurological deficits in mouse. This neuro-protection was associated by antiapoptotic activity of carvacrol characterized by decreased level of cleaved caspase-3 as a major marker of apoptosis (33). Similarly, Chen *et al.* demonstrated that neuroprotective effect of carvacrol was accompanied by its antiapoptotic effect mediated by inhibition of Ca^2+^-permeable, non-selective cation channel TRPM7. Increase of Bcl-2/Bax protein ratio and decrease of caspase-3 cleavage were also shown in their study (34). 

Recent studies demonstrated that in addition to apoptosis, dysregulation of autophagy can also contribute to cell death in cardiovascular diseases. Basically autophagy occurs at low level to maintain the cardiomyocytes homeostasis, but it is stimulated in response to pathological stimuli such as oxidative stress (35). Nakai *et al.* found that autophagy changes during different phases of pressure overload-induced cardiac hypertrophy. It was suppressed in hypertrophied hearts after aortic banding for 1 week, and upregulated in the failing hearts after 4 weeks; this suggests complicated regulatory effects of hypertrophic signalings on the phase-dependent change of autophagy (36). Ca^2+^/Calcineurin signaling pathway, which promotes cardiac hypertrophy by activating hypertrophic genes program, can inhibit cardiomyocyte autophagy in an AMPK-dependent manner (37). 

To the best of our knowledge*, *there is no report on the possible effect of carvacrol in regulation of autophagy process in cardiovascular system. Thus, there is a need for providing novel insight on cardioprotective effects of carvacrol. 

One of the factors contributing to the incidence of cardiac damage is the increased level of oxidative factors such as free radicals in the hypertrophied myocardial tissue. Oxidative stress contributes to the pathogenesis of hypertrophic cardiac dysfunction by affecting the ionic currents, structure, and performance of proteins, expression of genes, and apoptosis (4). Measurement of MDA can be an appropriate indicator of lipid peroxidation for assessing oxidative stress. Our results showed that in hypertrophied animals, serum concentration of MDA increased, while it decreased following the administration of carvacrol.

It has been shown that carvacrol can increase total radical-trapping antioxidant parameter in cultured cells. It is also able to remove nitric oxide radicals and inhibit lipids peroxidation leading to protective effects against oxidative stress (38). Canbek *et al.* reported that carvacrol increases glutation and catalase levels and decreases the MDA level in animals subjected to liver ischemia-reperfusion (39). Antioxidative effects of carvacrol in diethyl nitrosamine-induced hepatocellular carcinoma and in UVB-induced- lipid peroxidation lymphocytes in human were also demonstrated (40, 41). 

The study by Yu *et al.* showed that the intraperitoneal injection of carvacrol (25, 50, and 100 mg/kg) could decrease MDA level and simultaneously increase the antioxidative enzymes level (17).

DPPH is another indicator related to antioxidant status. In our study, it was observed that carvacrol inhibits DPPH free radicals in hypertrophied animals. These effects may probably be due to hydroxyl group on benzene cycle. Augmentation of antioxidant system is another mechanism that could be responsible for this effect. Future research can help to clarify this issue more.

In summary, our findings demonstrated that carvacrol decreases hypertrophy markers including blood pressure, HW/BW ratio, tissue fibrosis and ANP mRNA level in rats with abdominal aortic banding. Furthermore, carvacrol decreases cell size and ANP mRNA level in response to Ang II-induced hypertrophy in H9c2 cells. These effects were associated with antioxidant and anti-apoptotic effects of carvacrol. Consequently, due to the anti-hypertrophic and antioxidant effects of carvacrol, it could be considered as a therapeutic target in treating cardiac hypertrophy. 
